# Application of high-flow nasal cannula oxygen therapy in patient with pulmonary edema following cesarean-section under combined spinal-epidural anesthesia: A case report

**DOI:** 10.1097/MD.0000000000034140

**Published:** 2023-06-30

**Authors:** Taeil Lee, Helen Ki Shinn, Na Eun Kim, Doyeon Kim

**Affiliations:** a Department of Anesthesiology and Pain Medicine, Inha University, Incheon, South Korea; b Department of Anesthesiology and Pain Medicine, CHA Bundang Medical Center, CHA University School of Medicine, Seongnam, South Korea.

**Keywords:** cesarean-section, combined spinal-epidural technique, high-flow nasal cannula oxygen therapy, obstetric anesthesia, preeclampsia, pregnancy, pulmonary edema

## Abstract

High-flow nasal cannula (HFNC), which overcomes the disadvantages of the existing low flow mask, is an efficient method that can immediately provide a high volume of heated oxygen to the patient.^[1]^ Therefore, this case reports a case in which HFNC was applied to a pregnant with acute respiratory failure.

**Case:**

A 37-year-old woman pregnant (GA 30 + 5 weeks) with twin was diagnosed with preeclampsia. It was decided to perform an emergency Cesarean-section under combined spinal-epidural technique worsening respiratory failure. After delivery, maternal dyspnea was not alleviated applying of O_2_8 L/min via facial mask. Thus, high-flow nasal cannula (HFNC) oxygen therapy was applied (60 L/min, partial pressure of oxygen (FiO_2_) 80%) and SpO_2_ subsequently rose to 98% and the patient’s dyspnea was resolved.

**Conclusions:**

HFNC is a safe device that can effectively provide oxygen to pregnant with acute respiratory failure.

## 1. Introduction

Oxygen therapy is commonly provided as a low-flow system (e.g., nasal cannula or mask) or an intrinsic volume system (e.g., venturi mask or non-rebreather facemask).^[1]^ In contrast, high-flow nasal cannula (HFNC) is a unique form of noninvasive respiratory support therapy that delivers warmed/humidified inhalation rate (FiO_2_ 21–100%) oxygen at flow rates up to 60 L/min.^[2]^ This effect can improve mucosal function, airway cleaning, and patient comfort. It can also provide positive end-expiratory pressure to help expel carbon dioxide, reduce dead space, stretch collapsed alveoli, and ultimately improve tissue oxygenation.^[1]^ We reported a case of effective application of HFNC in pregnant woman underwent Cesarean section.

## 2. Case report

**Figure 1.**
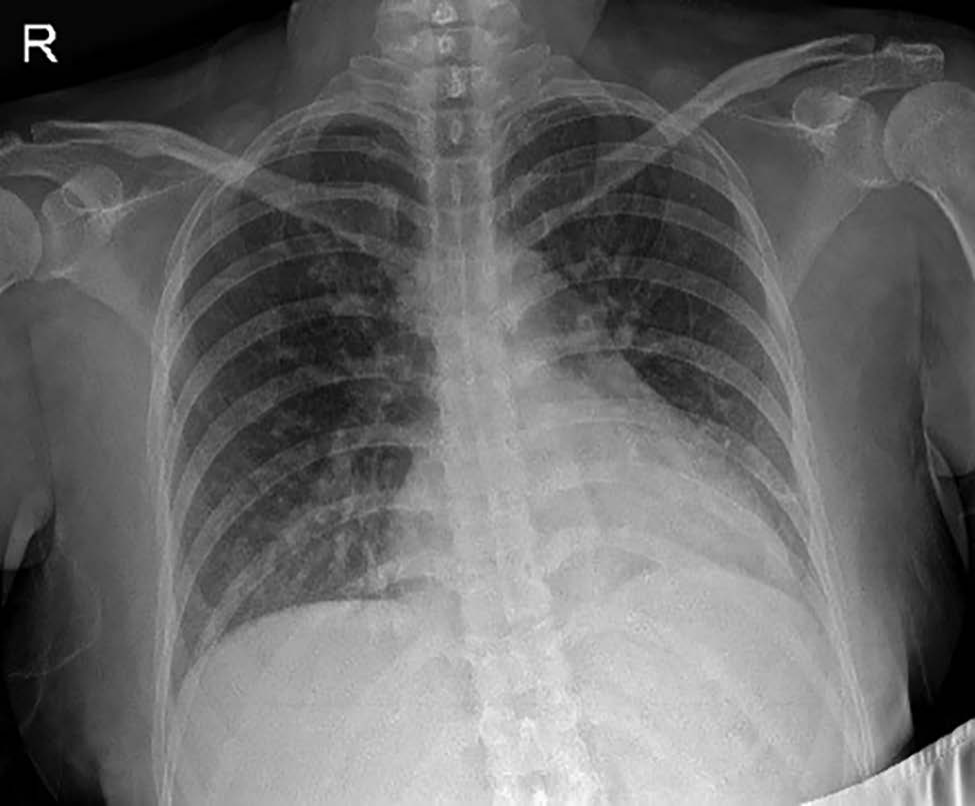
Chest X-ray at GA 30 + 5 weeks increased BLL haziness.

A 37-year-old first gravida pregnant woman visited the outpatient department of obstetrics and gynecology at 30 + 5 weeks of gestation age (GA). The patient was 172 cm in height and 82.5 kg in weight. She complained newly developed high blood pressure (BP) during pregnancy, facial edema, mild headache, and dyspnea from 2 weeks before visiting. She denied any medical history, including high blood pressure, before pregnancy. Her BP was 168/96 mm Hg and chest radiographs revealed haziness in both lower lung fields (Fig. 1). She was admitted to the obstetrics and gynecology department under the diagnosis of preeclampsia and started magnesium sulfate therapy.

**Figure 2.**
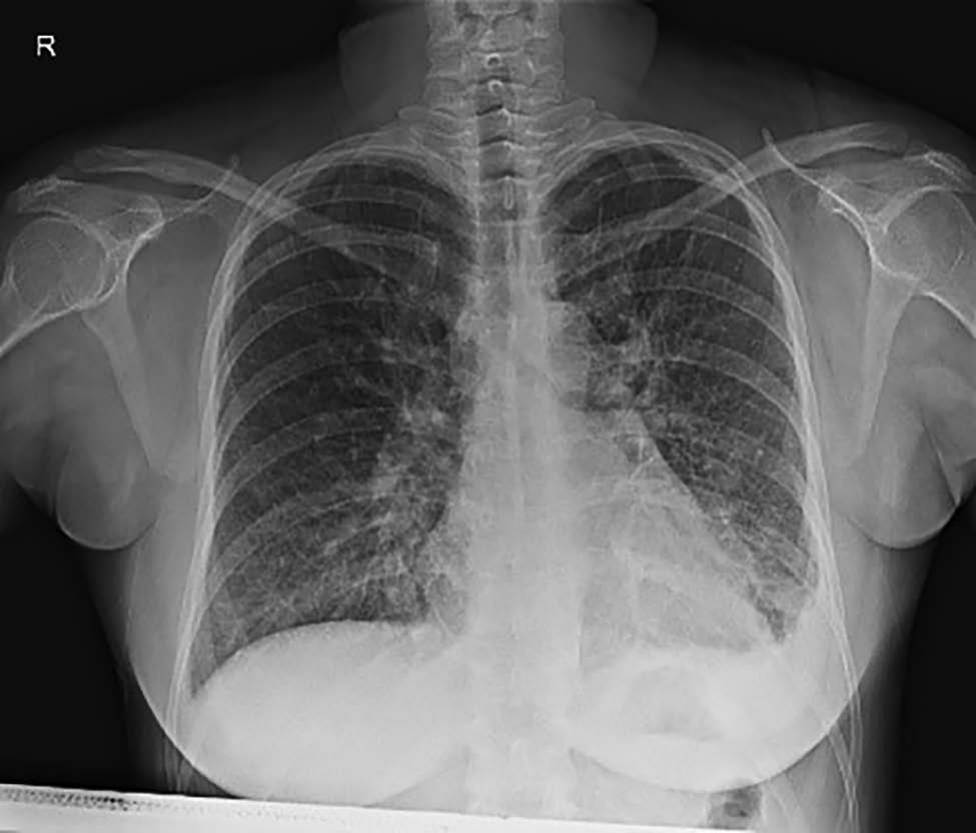
Chest X-ray at GA 36 weeks increased left pleural-effusion.

On the 3rd day of hospitalization (GA 31 weeks), follow-up chest radiographs demonstrated pleural effusion in left lower lung field (Fig. 2) and her urine output decreased. Although IV furosemide 20 mg have been added, there was no improvement in symptoms and chest radiographs. Thus, it was decided to perform emergency cesarean section at GA 31 + 3 weeks. Preoperative laboratory test was as follows: hemoglobin 7.5 g/dL, magnesium 4.0 mg/dL, calcium 7.6 mg/dL, and AST 65 IU/L. Electrocardiogram (ECG) showed normal sinus rhythm and anterior infarct.

### 2.1. Anesthetic management

No premedication was allowed. Magnesium sulfate was continuously infused. After entering operating room, standard monitoring, such as noninvasive noninvasive BP (NIBP), ECG, and pulse oximetry (SpO_2_), were applied. Initial vital signs were BP 210/121 mm Hg, heart rate (HR) 100 bpm, and SpO_2_ 95%. Combined Spinal-Epidural Anesthesia (CSE) was performed in the lateral decubitus position while supplying O_2_ at 5 L/min via facial mask. The double-space technique was used for CSE. First, after confirming the location of the epidural space at L 2–3 level, a catheter was mounted. Then, after confirming free flow of cerebrospinal fluid at L 3–4 level, 0.5% Bupivacaine 7.5 mg (Marcaine Heavy Injection 20 mg; Mitsubishi Tanabe Pharma Korea Ltd., Seoul, Korea) and Fentanyl 20 µg (Hana Fentanyl Citrate Inj., Hana Pharm Co., Ltd., Seoul, Korea) were administered intrathecally. To prevent spinal anesthesia induced hypotension, 500 mL of colloid (Volulyte^®^, Fresenius Kabi Korea Ltd., Seoul, Korea) and prophylactic phenylephrine were infused (30 mg/h). Even after anesthesia, the patient showed a consistently high BP of 198/88 mm Hg.

During the CSE technique, the patient continued to express of mild dyspnea and SpO_2_ was maintained at 87% to 95%. Thus, oxygen flow rate was increased to 8 L/min. After the CES technique was completed, the patient’s posture was changed to the supine position with left tilting to prepare for surgery. The patient expressed aggravated dyspnea and SpO_2_ decreased dramatically to 79% to 80%. Thus, oxygen flow rate was increased to10 L/min and HFNC (Optiflow™ nasal high flow therapy, Fisher & Paykel Healthcare Inc, Auckland, New Zealand) was applied to supply proper oxygen to the patient and fetus (60 L/min, FiO_2_ 80%). After confirming of the sensory blockade level had reached T4, surgery was started. In addition, arterial line was placed on the left radial artery for continuous monitoring of arterial blood gas. A 37-year-old twin-pregnant woman presented with newly developed high blood pressure at gestational age (GA) 30 + 5 weeks. Arterial blood gas analysis (ABGA) revealed PaO_2_ 51.6 mm Hg and O_2_ saturation 81.8%. During management for dyspnea relief, fetuses were delivered (at 5 minutes after initiation of surgery). Information of newborns were as follows: 1 minute to 5minute APGAR score, 1st baby (1.8 kg): 2 and 6; 2nd baby (1.57 kg): 6 and 8, respectively. After delivery of the fetuses, oxytocin 10 U with lactated ringer’s solution was infused continuously. In addition, Carbetocin 100 µg and Sulprostone 1000 µg were administered intravenously to minimize blood loss and to produce uterine contraction. The patient’s BP decreased to 110/50 mm Hg and dyspnea was improved. The flow rate of HFNC therapy was gradually reduced to 6 L/min. ABGA results were PaO_2_ 81.1 mm Hg SpO_2_ 98%.

At the end of surgery (20 min after application of HFNC therapy), V/S were BP 122/66 mm Hg, HR 67 bpm, and SpO_2_ 96%. PaO_2_ increased to 60.5 mm Hg. The duration of anesthesia was 75 minutes and the duration of surgery was 60 minutes. A total of 1000 mL of crystalloid and 500 mL of colloid were administered and an estimated blood loss was 800 mL. She was transferred to the sub-ICU in the general ward. HFNC therapy was maintained as 60 L/min FiO_2_ 0.8 was applied and the patient.

### 2.2. Postoperative progress

**Figure 3.**
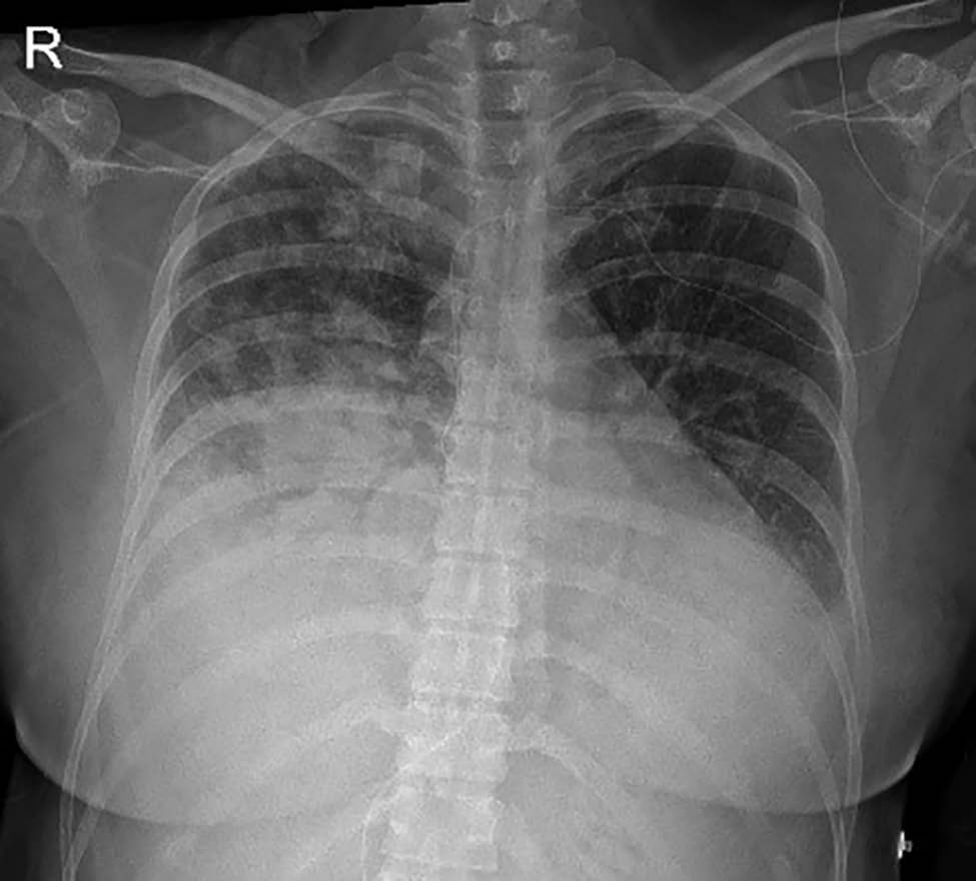
Chest X-ray POD 1 decreased RUL haziness, remaining haziness at RUL. POD = postoperative day.

On postoperative day (POD) 1, HFNC 50 L/min FiO_2_ 0.5 was applied and the patient expressed improvement in respiratory distress. V/S were stable: BP 137/95 mm Hg, HR 80 bpm, RR 25 per minute. ABGA showed improved oxygen status: PaO_2_ 230 mm Hg and O_2_ saturation 99%. Magnesium sulfate therapy (40 mL/h) was continued. Follow-up chest radiographs showed decreased amount of pleural effusion (Fig. 3). EKG showed septal infarct and lateral ischemia. Thus, consultation with a cardiology department was requested and cardiac markers were examined: NT-proBNP 5235 pg/mL, CK-MB 11.3 ng/mL, and Troponin-I 2.470 ng/mL, respectively. After performing transthoracic echocardiogram, stress induced cardiomyopathy with mild left ventricular dysfunction were diagnosed. To maintain the negative intake/output balance, loop diuretics were continuously administered. For postoperative analgesia, intravenous patient-controlled analgesia (ANAPA AC0605^®^; Ehwa Biomedics, Korea) was applied with fentanyl 1000 µg + ropivacaine 300 mg.

**Figure 4.**
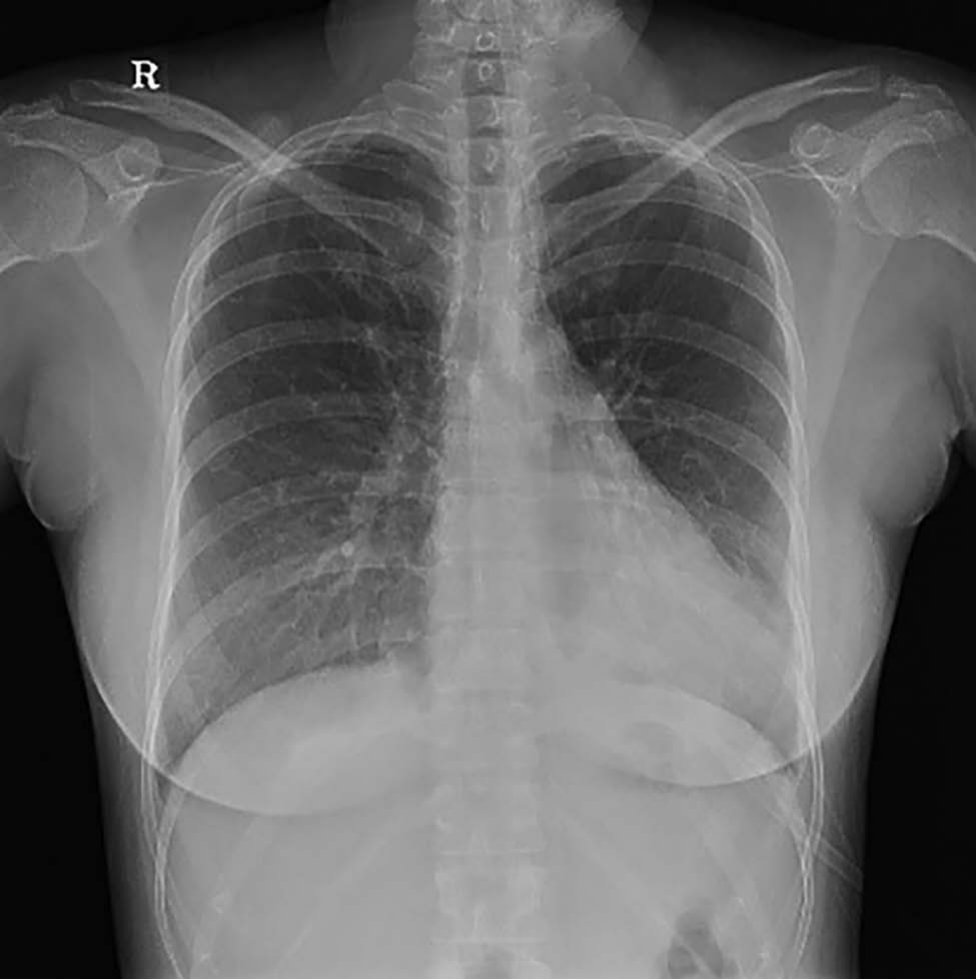
Chest X-ray POD 4 decreased BLL haziness, but remained. POD = postoperative day.

On POD 4, the patient did not complain respiratory distress. HFNC therapy was tapered and discontinued. SpO_2_ was 98% in room air and no dyspnea was observed. On POD 5, the patient did not show any symptoms of dyspnea in room air. ABGA revealed improved oxygen status: PaO_2_ 92.1 mm Hg and O_2_ saturation 97%. Improvements in pleural effusion and haziness were observed in chest radiographs (Fig. 4). The patient was discharged.

On outpatient follow-up (POD 29), TTE revealed the restored LV systolic function. The patient no longer complained of dyspnea and all medications were discontinued.

All studies were conducted after the patient’s informed consent was written and the In-Ha institutional review board (IRB) review was passed (IRB No. 2023-02-007).

## 3. Discussion

This case demonstrated the successful use of HFNC therapy for respiratory management of a patient with persistent dyspnea and hypoxemia after Cesarean delivery (Table 1). With rapid application of HFNC therapy, dyspnea was resolved and the patient’s oxygen status progressively improved. Our patient was diagnosed with preeclampsia. Moreover, based on the results of postoperative cardiac markers and echocardiography, her respiratory distress was considered to be of cardiovascular origin.^[2]^ Thus, we realized that HFNC therapy was the useful respiratory management tool for cardiovascular maintained until resolution of dyspnea and pulmonary effusion. Finally, the patient could discharge without invasive procedure or further complications.

**Table 1:** HFNC therapy setting and oxygen status over time.

	Before HFNC	10 min after applying HFNC	POD 1	POD 2	POD 3
HFNC setting		FiO_2_ 0.8, 60 L/min	FiO_2_ 0.5, 50 L/min	FiO_2_ 0.5, 50 L/min	FiO_2_ 0.3, 25 L/min
pH	7.36	7.39	7.462 ↑	7.534 ↑	7.499 ↑
PaCO_2_ (mm Hg)	44.8	41.4	35.4	39.6	41.6
PaO_2_ (mm Hg)	51.6↓	81.1↓	230.0 ↑	159.0 ↑	136.0 ↑
HCO_3_^−^ (mmol/L)	25.1	24.9	25	33 ↑	32 ↑
SpO_2_ (%)	81.8	98	99	100	99
Chest radiograph	Increased right lung haziness	Decreased RUL haziness			Decreased BLL haziness
Symptom	Respiratory distress	Improvement in respiratory distress			

HFNC **=** high-flow nasal cannula, POD **=** postoperative day.

HFNC therapy can be highly useful in clinical practice. The mechanism of HFNC is summarized in Table 2. Heated and humidified oxygen provided by HFNC have many advantages including the improved secretion clearance, increased the respiratory efficacy by washing out the dead space, and reduced airway inflammation and energy consumption.^[3]^ In addition, very high gas flow reduces inspiratory demand to meet the patient’s inspiratory flow needs^[4]^ and increase functional residual capacity or end-expiratory lung volume.^[5]^ Furthermore, patient’s compliance is also high because high flow rates of oxygen can be administered in a comfortable state without invasive procedures.

**Table 2:** The mechanism of HFNC therapy.

Mechanisms of HFNC
• Heated and Humidified Oxygenation
• Reduced Inspiratory Demand
• Positive end-expiratory pressure effect
• Increased patient compliance
• Determined Oxygen Concentration Delivery
• Washout of Dead-space

HFNCM = high-flow nasal cannula.

To apply HFNC therapy, 2 parameters must be set: the inspiratory air flow rate and the FiO_2_. Typically, the flow rate is initially set to 20 to 35 L/min (range 5–60 L/min), then the FiO_2_ to achieve the desired peripheral site oxygen saturation (21–100% range). Both increase of flow rate and FiO_2_ have the effect of improving the oxygen saturation of peripheral sites. If the respiratory rate does not stabilize after applying the device to the patient, or if oxygenation does not improve adequately and difficult respiratory efforts continue to be maintained, the flow rate can be increased by 5 to 10 L/min increments. In general, maximizing flow rate is recommended first to keep FiO_2_ below 60%, but you can also try increasing FiO_2_ for proper oxygenation. HFNC can be applied long-term for several days, with a gradual decrease in flow rate and FiO_2_ as respiration improves, and when the flow rate reaches ≤20 L/min and FiO_2_ reaches ≤50%, it can be switched to the traditional low flow rate nasal cannula system.

HFNC indications are primarily subjective rather than absolute. Common indications for adults are listed in Table 3. In addition to these indications, clinical practice may use HFNC in a variety of situations. In this case, we managed the patient’s airway without tracheal intubation. Because tracheal intubation and accompanying neuromuscular blockade and mechanical ventilation could contribute to the occurrence of postoperative maternal pulmonary complications. Thus, to maintain the patient’s oxygen status safely, we applied HFNC therapy. Previous study reported the case which used HFNC therapy to the obstetric patient with interstitial pneumonia prior to delivery.^[11]^ However, to the best of our knowledge, there has been no report on the effective application of HFNC therapy during Cesarean-section. As we know, airway management is difficult in the obstetric patient due to the various factors including vascular and edematous mucosa, decreased functional residual capacity and increased oxygen requirements, reduced lower esophageal sphincter tone, and enlarged breasts.^[12]^ Thus, appropriate application of HFNC therapy can improve oxygenation without tracheal intubation in obstetric patients. However, when using HFNC, there may be a risk of nosebleeds and gastric aspiration due to swelling of the nasopharyngeal mucosa, so care must be taken when applying to pregnant women.

**Table 3:** Indication for HFNC therapy.

HFNC indications
Acute Hypoxemic Respiratory Failure (Mainly from Community-acquired Pneumonia)^[6]^
To maintain oxygen in patients at low risk of reintubation after extubation^[7]^
It can be used for pre-oxygenation before intubation, but in high-risk surgery with a high complication rate such as hypoxemia, hypotension, and even cardiac arrest^[8]^
Do Not Resuscitate (DNR)/Do Not Intubate (DNI) in Respiratory Distress Patients with acute hypoxemia and mild hypercapnia (pCO2 < 65)^[9]^
To improve the severity of dyspnea in patients with Cardiogenic Pulmonary Edema^[10]^

HFNC = high-flow nasal cannula.

In conclusion, HFNC therapy was a useful respiratory management method that reduces the need for intubation. It showed the ability to reduce cardiovascular-induced dyspnea and hypoxemia.

## Author contributions

**Conceptualization:** Doyeon Kim.

**Investigation:** Helen Ki Shinn.

**Methodology:** Helen Ki Shinn.

**Supervision:** Na Eun Kim.

**Validation:** Na Eun Kim.

**Visualization:** Na Eun Kim.

**Writing – original draft:** Taeil Lee, Doyeon Kim.

**Writing – review & editing:** Taeil Lee, Doyeon Kim.
